# Improving osteoporosis treatment rates in inpatients admitted with hip fracture: A healthcare improvement initiative in a tertiary referral hospital

**DOI:** 10.1002/agm2.12229

**Published:** 2022-11-15

**Authors:** Andrew Gan Lin, Nargis Shaheen, Kirtan Ganda, John Cullen, Louise M. Waite, Markus J. Seibel

**Affiliations:** ^1^ Department of Endocrinology Concord Hospital Concord New South Wales Australia; ^2^ Centre for Education and Research on Ageing (CERA), Department of Geriatric Medicine Concord Hospital Concord New South Wales Australia; ^3^ Concord Clinical School, Faculty of Medicine and Health University of Sydney Sydney New South Wales Australia

**Keywords:** hip fracture, inpatient protocol, osteoporosis, quality improvement

## Abstract

**Objective:**

This healthcare improvement initiative was designed to increase inpatient osteoporosis treatment after hip fracture.

**Methods:**

A new protocol was developed by Geriatric Medicine and Endocrinology departments at a tertiary care hospital in Sydney. Its aim was to standardize assessment and treatment of osteoporosis in patients admitted with hip fracture. Eligible inpatients would receive intravenous zoledronic acid during their admission. A 6‐month sample of hip fracture patients admitted after the protocol's implementation was compared to a group admitted before. Data collected included demographics, biochemistry, treatment rates, adverse effects, and admission survival.

**Results:**

There was a considerable increase in osteoporosis treatment after introducing the protocol. Before the protocol's introduction, none of 36 eligible patients received treatment. After the intervention 79% (23 out of 29) of eligible patients were treated.

All treated patients had renal function and serum calcium levels checked post‐infusion with no adverse outcomes. Eight patients developed flu‐like symptoms within 24 h of the infusion. There were no instances of arrhythmias, ocular inflammation, or death. The cost per patient treated was AUD $87.

**Conclusion:**

Adopting a standardized protocol for osteoporosis treatment in patients admitted for hip fracture was effective in improving treatment rates whilst being relatively safe and inexpensive.

## Graphical abstract

Our hospital introduced a standardized protocol for the treatment of osteoporosis in patients admitted for hip fracture. The aim of this article was to describe its implementation as well as assess the protocol's effect on treatment rates and safety. In our analysis, there was significant improvement in treatment rates with no instances of acute kidney injury, hypocalcaemia, or inpatient death. The protocol triggers a simple and inexpensive intervention that can easily be implemented.

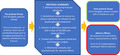

## 1 INTRODUCTION

Hip fractures are among the most significant consequences of untreated osteoporosis. In 2015–2016, the Australian Institute of Health and Welfare reported 18,746 hip fractures across Australia with significant effects on life expectancy, mobility, and independence.^1^ Five percent of patients do not survive their initial hospital admission and up to 20% die within a year following hip fracture.^2^ At least 10% of patients are newly discharged to an aged care facility after their admission,^3^ and 12 months post fracture half of all patients still have ongoing mobility deficits.^4^

The cost of hip fracture to the Australian healthcare system is substantial and includes not only the direct cost of fracture care but lost productivity, ongoing rehabilitation, day to day assistance, and residential aged care facility placement. In 2012, the total cost attributed to osteoporosis and fractures in Australia was approximately $2.7 billion with the costs of hip fractures alone estimated to be $790 million.^5^

Any osteoporotic fracture portends an increased risk for future fractures. After a hip fracture, the risk of any further fracture increases 2.5 times in women, while for men the risk is almost five times greater than for those without hip fractures.^6^ Bone‐protective therapies such as bisphosphonates reduce the risk of subsequent fracture and are considered standard care post minimal trauma hip fracture.^7^ Their benefits have been well established; in women over 70 years of age with previous vertebral fractures risedronate reduced hip fracture rates by 60%.^8^ Subsequently, Lyles and colleagues showed zoledronic acid administered within 90 days of a hip fracture reduced the risk of new fractures by 35% and decreased all‐cause mortality by 28%.^9^

Despite the clear evidence for the effectiveness of anti‐resorptive therapy, osteoporosis is still under diagnosed and under ‐treated. In 2007, Teede and colleagues reported that 10% of patients admitted for minimal trauma fracture received appropriate investigation and <10% were commenced on bone protective therapies in the inpatient setting.^10^ Similarly, in a survey of 69,000 post‐menopausal women with a history of fracture, Eisman and colleagues found only 28% were prescribed any specific therapy for osteoporosis.^11^

A decade later, the osteoporosis treatment gap remains a significant problem. In 2018, the Australian & New Zealand Hip Fracture Registry (ANZHFR) estimated that only 35% of patients were taking bone‐protective medications at 120 days after discharge.^4^ That year, <10% of patients admitted for hip fracture to our hospital received bone‐protective treatment prior to discharge. Instead of a systemized approach to osteoporosis for inpatients, our previous recommendation was for the patient's general practitioner to initiate anti‐resorptive therapy post discharge. However, given the high risk of further fractures and the generally low treatment rates in these patients, a more proactive approach was required. We therefore introduced a new inpatient treatment protocol in April 2019 that aimed to deliver treatment for osteoporosis to all eligible patients during their admission for an incident hip fracture. Here we report, according to SQUIRE2.0 guidelines,^12^ the implementation, outcomes, and safety of this new protocol.

## 2 METHODS

### 2.1 Design of protocol

The protocol was developed as a collaboration between the Geriatric Medicine and Endocrinology departments at a tertiary care hospital in metropolitan Sydney. We are a public teaching hospital with links to a local university. Implementation of the protocol was led by a consultant geriatrician supported by the orthopedic, pharmacy, and nursing departments.

In our institution, hip fracture patients are routinely admitted to the orthopedic service and automatically referred to the ortho‐geriatric service for consultation. There is a sole geriatrician responsible for the service and assessing each patient's suitability for the protocol. The geriatrician or her team of junior medical staff would then prescribe zoledronic acid and cholecalciferol per the protocol as well as monitor the patient for adverse clinical or biochemical effects. Our hospital uses electronic medical records and prescribing software, but there were no prompts embedded into this system. Given all patients with hip fractures contribute to our local database for submission to the Australian and New Zealand Hip Fracture Registry, this protocol was considered to be part of the ortho‐geriatric's service routine care.

In Australia, junior hospital doctors rotate through different hospital units and services multiple (usually more than four) times a year. The protocol was introduced during the orthopedic term orientation sessions. This typically included four junior medical staff every 10 weeks. An education session was delivered by the geriatrician leading the orthogeriatric service and covered the protocol's rationale, use, and common side effects of zoledronic acid.

The endocrinology department provided education to orthopedic nurses around drug safety, administration, and monitoring. These were integrated as part of a larger program of regular teaching sessions held weekly. The pharmacy department was responsible for medication dispensing and monitoring. Junior medical staff were trained in osteoporosis assessment, drug administration, and monitoring for adverse effects. They also provided patient education and written resources.

### 2.2 The protocol

**FIGURE 1:**
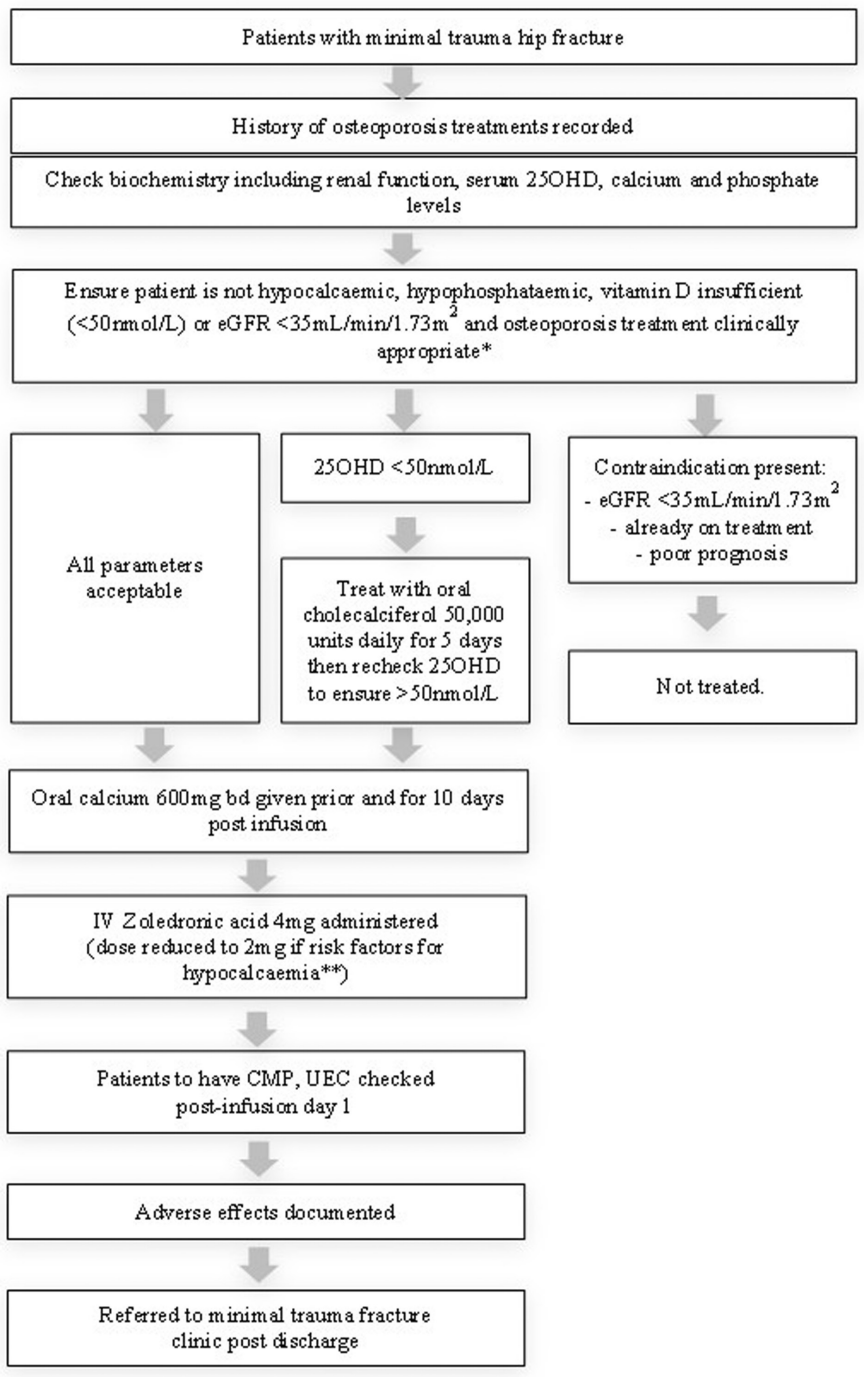
Summary of the inpatient osteoporosis treatment protocol. *Including consideration of comorbidities and prognosis. **Patients with renal impairment, BMI ≤ 14 kg/m^2^, or malnutrition. 25OHD, 25hydroxy‐vitamin D; IV, intravenous.

The protocol (Figure 1) aimed to provide eligible patients with anti‐resorptive treatment during their admission for hip fracture. A history of prior osteoporosis treatment was documented. Patients with minimal trauma hip fractures had routine biochemistry at admission including renal function, serum calcium, phosphate, and 25‐hydroxyvitamin D levels. Patients were considered eligible for treatment with zoledronic acid if they were not currently receiving osteoporosis therapy; had sufficient renal function (estimated glomerular filtration rate [eGFR] ≥ 35 ml/min/1.73 m^2^); and serum calcium and phosphate levels within the reference range without evidence for metabolic bone disease. The attending geriatrician assessed the patient's eligibility for treatment based on comorbidities or prognostic considerations. Patients with vitamin D insufficiency (25‐hydroxyvitamin D < 50 nmol/L) were provided with supplementation in the form of oral cholecalciferol 50,000 units daily for 5 days. Zoledronic acid 4 mg was then administered by intravenous infusion. Post infusion vitamin D, calcium levels, and adverse events were documented.

### 2.3 Assessment of protocol implementation, outcomes, and safety

Two groups of patients were compared for this analysis: A cohort of patients admitted prior to protocol (April to June 2018), and a second cohort the same size admitted after the implementation of the protocol (from January 2020 onwards). Cases were identified for review by a search of disease‐related groupings for fractures involving the intracapsular and intertrochanteric regions of the femur as well as cases submitted to the Australian and New Zealand Hip Fracture Registry. Cases were excluded if the mechanism of hip fracture was not consistent with minimal trauma or if patients were initially admitted to another hospital for treatment of the hip fracture.

Data for the pre‐protocol group was collected retrospectively from the electronic medical record, including patient age, sex, usual place of residence, use of bone‐protective medications before and during the admission, comorbidities, and inpatient death. Estimated glomerular filtration rate, serum creatinine, corrected serum calcium, phosphate, and 25‐hydroxyvitamin D levels were also recorded. In the post‐protocol group, the same data were collected prospectively, including repeat serum 25‐OH vitamin D levels after cholecalciferol replacement. In patients who received zoledronic acid, adverse reactions were recorded along with post‐infusion monitoring of renal function and serum calcium levels. If renal impairment or hypocalcaemia was detected, patients were reviewed to determine whether this was due to the intervention.

Demographic characteristics, blood biochemistry, and therapy prior to admission were compared between the pre‐ and post‐protocol groups, and within the post‐protocol group, between patients who received receive zoledronic acid versus those who did not. Chi‐square test was used for categorical variables and Student's *t*‐test or Mann‐Whitney U tests for continuous variables.

The findings presented constitute part of routine clinical audit undertaken by the hospital as part of healthcare improvement. The project was reviewed by the (blinded) Local Health District – (blinded) Human Research Ethics Committee and was granted an exemption from further ethical review since it complied with section 5.122 and 5.1.23 of the National Statement on Ethical Conduct in Human Research (2007).

## 3 RESULTS

### 3.1 Pre‐protocol group

Baseline characteristics of the study population are shown in Table 1. Over 80% of patients in the pre‐protocol group were not taking bone‐protective medications prior to admission, while 9 patients (17%) received either denosumab (*n* = 4), oral bisphosphonate (*n* = 4), or intravenous bisphosphonates (*n* = 1). Of the 43 patients without pre‐admission bone‐protective therapy, review of biochemistry and patient records indicated that treatment would have been contraindicated in seven of these patients due to impaired renal function (*n* = 5), hypocalcaemia (*n* = 1), or other reasons (*n* = 3). Therefore, 36 patients were eligible for bone‐protective treatment although none received such treatment before discharge.

**TABLE 1:** Baseline characteristics of the pre‐protocol and post‐protocol groups.

Variable	Pre‐protocol group (*n* = 52)	Post‐protocol group (*n* = 52)	*p*‐Value
Median Age (IQR)	87 years (75–90)	85 years (78–93)	0.755
Female (*n*; %)	38 (73.1)	38 (73.1)	1
Residential Aged Care Facility pre‐admission (%)	14 (26.9)	10 (19.2)	0.352
Median eGFR in ml/min/1.73 m^2^ (IQR)	71 (47–84)	69 (43–79)	0.270
Number of patients with eGFR <35 ml/min/1.73 m^2^ (%)	5 (9.6)	11 (21.2)	0.103
Average Vitamin D level on admission in nmol/L (SD)	68 (28)	62 (27)	0.288
Vitamin D insufficient on admission [<50 nmol/L] (%)	10 (19.2)	15 (28.8)	0.199
Mean serum corrected calcium in mmol/L^a^ (SD)	2.43 (0.13)	2.23 (0.16)	<0.001
Bone protective medications prior to admission (%)	9 (17.3)	7 (13.4)	0.587
Number eligible for treatment per protocol	36 (69.2)	29 (55.8)	0.156

^a^ Calcium reference range: 2.10–2.60 mmol/L.

### 3.2 Post‐protocol group

Baseline characteristics are again summarized in Table 1. Compared to the pre‐protocol group, mean serum corrected calcium was slightly lower in the post‐protocol group, with all other parameters being balanced between both groups. Seven patients were already receiving bone‐protective therapy (intravenous bisphosphonates, *n* = 2; oral bisphosphonates, *n* = 3; denosumab, *n* = 2), and 16 patients were ineligible due to a combination of poor renal function (*n* = 11), poor prognosis (*n* = 3), or inpatient death (*n* = 7).

**FIGURE 2:**
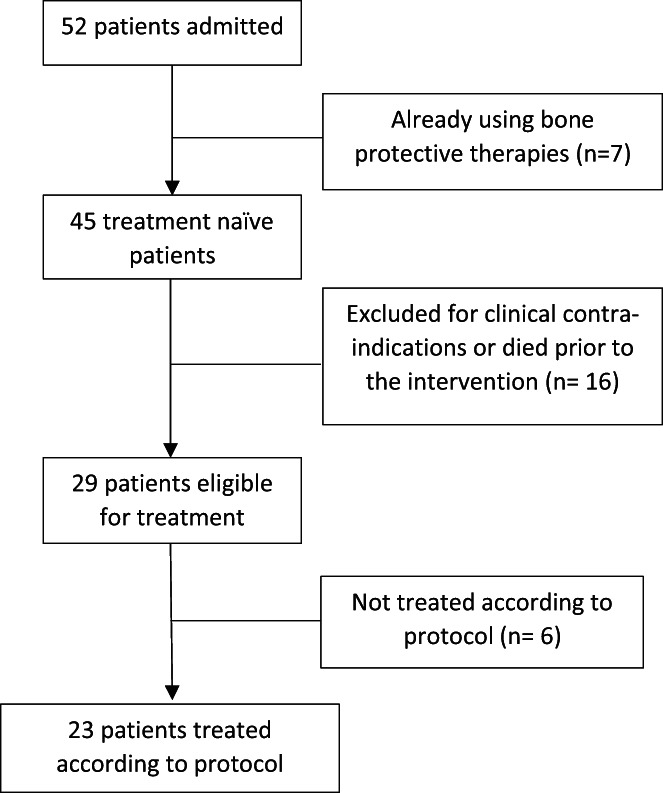
Eligibility and treatment of post‐protocol group

Six eligible patients (21%) did not receive zoledronic acid: one patient declined, two were known to an external physician who recommended deferring treatment, one patient was transferred to another facility, and two patients required further investigation. A total of 23 (79%) eligible patients received zoledronic acid as inpatients (Figure 2). Patients who did not receive zoledronic acid had a lower median eGFR (54 vs. 74 ml/min/1.73 m^2^, *p* = 0.008) but did not differ in any other variable (Table 2).

**TABLE 2:** Characteristics of treated and untreated patients after the introduction of zoledronic acid protocol.

Variable	Treated (*n* = 23)	Nontreated (*n* = 29)	*p*‐Value
Median age in years (IQR)	84 (78–94)	87 (77–90)	0.412
Female gender (*n*;%)	16 (69.6%)	22 (75.9%)	0.611
Residential aged care facility pre‐admission (%)	3 (13.0%)	7 (24.1%)	0.313
Median eGFR in ml/min/1.73 m^2^ (IQR)	74 (66–84)	54 (25–75)	0.008
Average vitamin D level on admission in nmol/L (SD)	62 (25)	62 (29)^a^	0.995
Vitamin D insufficient on admission [<50 nmol/L] (%)	7 (30.4%)	8 (33.3%)^a^	0.831
Mean serum corrected calcium in mmol/L^a^ (SD)	2.22 (0.13)	2.23 (0.19)	0.721

^a^ *N* = 24 (due to five patients with no vitamin D level performed).

### 3.3 Monitoring and safety post zoledronic acid infusion

In the post‐protocol group, all treated patients had calcium and renal function monitored on day one post infusion. No patient developed hypocalcaemia and no instances of acute renal impairment (according to KDIGO criteria; i.e., an increase in serum creatinine >26.5 μmol/L) were detected. Seven patients with vitamin D deficiency (<50 nmol/L) at baseline received supplementation with 50,000 units of oral cholecalciferol daily for 5 days. In all of these patients, serum 25‐hydroxy vitamin D level improved from a median 37 nmol/L (IQR 20‐44 nmol/L) to a median of 59 nmol/L (IQR 52‐70 nmol/L). Eight patients (35%) developed fevers and/or myalgias within 24 h of zoledronic acid infusion. There were no instances of less common adverse effects such as cardiac arrhythmias, ocular inflammation, or death post‐treatment.

### 3.4 Protocol Implementation

We followed the protocol closely in the majority of patients. Exceptions to this included six patients mentioned above who did not have treatment despite their eligibility. Deferring treatment as per the protocol occurred when the geriatrician exercised clinical judgment or discussed treatment with a patient's existing specialist. In two cases the geriatrician favored further investigations prior to initiating bisphosphonate treatment (such as suspecting metabolic bone disease) and in another two cases treatment was deferred to post discharge at the request of the patient's specialist.

### 3.5 Cost

The cost of 5 days of high dose oral cholecalciferol and one dose of intravenous zoledronic acid 4 mg was 87 Australian dollars per patient. Most of the laboratory testing was part of routine clinical care, including renal function, serum calcium and vitamin D levels.

## 4 DISCUSSION

### 4.1 Effectiveness of a standardized inpatient osteoporosis protocol

Osteoporosis treatment rates are a key benchmark advocated by the Australian and New Zealand Hip Fracture Registry. Prior to implementing our protocol, inpatient osteoporosis treatment was provided at the discretion of individual hospital physicians. Most commonly, this would be deferred as a follow‐up recommendation with the patient's general practitioner. Among our group of geriatricians and endocrinologists, we observed the majority of returning patients would not have this addressed. Therefore the consensus was to create a standardized protocol for inpatient osteoporosis treatment.

Adopting our protocol decidedly improved inpatient osteoporosis treatment rates. Within 2 years of introducing the new protocol, the proportion of patients receiving bone‐protective medication prior to discharge improved from <5% to 60%. Patients not suitable for protocol treatment were referred for individualized investigations and treatments post discharge.

The improved prescription rate is comparable to a previously reported quality improvement program by Kuiper and colleagues who primarily administered zoledronic acid on the day of discharge.^13^ Their intervention included delivering a teaching program to residents and creating a standardized admission order set for investigations and treatment with zoledronic acid. Within one cycle of their audit the authors reported increased treatment rates from zero to 57% (25/44) of eligible inpatients. Although adverse effects were not reported, there were no re‐admissions for acute kidney injury or hypocalcaemia.

There are many barriers contributing to the osteoporosis treatment gap including cost, adherence to therapy, and the logistical difficulties of investigation and treatment. A further issue is that different settings adopt diverse models of care (utilizing a combination of ortho‐geriatric and fracture liaison services) usually driven by available resources.^14^ Our hospital's protocol was predominantly implemented by a specialized ortho‐geriatric team led by a consultant geriatrician, where all hip fracture patients were reviewed by this physician.

The protocol had several important benefits including supporting adherence by administering a yearly treatment, not delaying treatment for nonessential investigations such as bone densitometry, and allowing osteoporosis follow‐up to be deferred for months if necessary. Thus, treatment during the acute admission ensured there was no potential for delaying anti‐resorptive therapy due to delayed or missed follow‐up. The long‐lasting effects of intravenous bisphosphonates creates a wider timeframe that allows for further investigations and other management options as required. Using intravenous bisphosphonates also improves patient adherence over oral therapy. For example, the World Hip Trauma Evaluation (WHiTE) cohort study observed only a third of the 2853 patients prescribed oral bisphosphonates remained adherent at 120 days.^15^ Lastly, the implementation of an inpatient treatment protocol required minimal additional staffing or resources compared to alternatives such as employing a fracture liaison coordinator.

### 4.2 Monitoring and safety

All treated patients were monitored for renal dysfunction post infusion with no cases of acute renal impairment. The literature suggests such an event would be relatively uncommon and our study was therefore not powered sufficiently to identify infrequent adverse events. The HORIZON Pivotal Fracture Trial (PFT) and HORIZON‐RFT trials found no significant difference in renal adverse events between zoledronic acid and placebo group.^9^, ^16^ HORIZON‐PFT found no bisphosphonate associated long‐term renal function deterioration beyond the age‐related changes seen in both groups. In the HORIZON‐Glucocorticoid Induced Osteoporosis trial involving 833 patients reported two cases of acute renal failure in the risedronate group and one in the zoledronic acid group—all were considered related to underlying diseases. Based on post‐marketing data the risk factors for acute renal impairment include underlying moderate to severe renal impairment, severe dehydration, and use of diuretic medication.^17^

In our clinical experience, hypocalcemia is occasionally observed after zoledronic acid infusion and this is more common in underweight or malnourished patients. This was the rationale for including both routine calcium supplementation as well as zoledronic acid dose reduction in these patients. The HORIZON PFT trials prescribed all patients supplementation of 1000–1500 mg calcium and vitamin D 400–1200 units daily.^16^

Serum calcium levels were measured in all our patients post zoledronic acid infusion with no cases of hypocalcemia observed. In HORIZON‐PFT, hypocalcemia affected 1.3% of 3800 women treated with intravenous zoledronic acid and usually occurred between 9 and 11 days post‐infusion and was transient and asymptomatic. Ideally, future iterations of the protocol should include an additional calcium check in the second week post infusion.

### 4.3 Efficacy of protocol's vitamin D replacement

Vitamin D deficiency is a contraindication for therapy with potent anti‐resorptive drugs and often leads to delays in treatment initiation, particularly if low doses of cholecalciferol are being used to correct it. In our study, seven patients eligible for treatment with zoledronic acid were found to be vitamin D deficient on admission. In all of these patients, high dose oral cholecalciferol (50,000 units for 5 days) was adequate to achieve 25‐hydroxy vitamin D levels above 50 nmol/L. Similarly, in the HORIZON‐PFT trial, subjects with vitamin D levels below 37.5 nmol/L received a loading dose of 50,000–125,000 units of cholecalciferol 14 days before being treated with zoledronic acid. Thereafter all patients received daily oral calcium (1000–1500 mg) and vitamin D (800–1200 units).^9^ Hence, short‐term high‐dose vitamin D replacement therapy represents an effective and safe strategy for the rapid normalization of vitamin D levels in deficient patients.

### 4.4 Cost of protocol implementation

The prescribing cost at our hospital was 87 Australian dollars per patient treated, plus minor cost for laboratory testing, most of which was part of routine clinical care. Given the known efficacy of bisphosphonates in the setting of secondary prevention of fracture we believe this protocol has the potential to be extremely cost effective in terms of preventing future fractures with their attendant suffering, disability, and healthcare costs in readmission and treatment.

### 4.5 Limitations

Results from the pre‐protocol (2018) group were gathered retrospectively from medical records. In this sample, recording of osteoporosis medications was reliant on self‐report and accurate documentation which may have led to under‐reporting. Another limitation was that safety and adverse effects were only assessed during the patient's admission. Post discharge adverse effects including hypocalcemia or ocular side effects may have been missed. A follow‐up phone call from a consultant geriatrician will be included in the next iteration of our protocol. Finally, denosumab was not utilized in this protocol; however, future versions of the protocol could incorporate this medication, particularly in those with significant renal impairment.

At the time of admission, the diagnosis of osteoporosis was based on the presence of a major minimal trauma fracture to the hip ascertained by clinical history and x‐ray. Hip fractures in elderly persons (median age in our study was 86 years) are almost always due to osteoporosis. At the time of admission and surgery, it was impractical to perform a DEXA scan for these patients. However, all survivors are seen at the outpatient fracture Liaison Service 12 months after their admission. At this point, most patients receive a DEXA scan and are assessed for further fractures and falls as well as quality of life, mobility, and the ability to perform ADLs. These data are currently being collected as part of a larger research study.

## 5 CONCLUSION

The use of a standard treatment protocol for osteoporosis in patients admitted for hip fracture was both safe and effective. The protocol triggers a simple and inexpensive intervention that resulted in a marked improvement in osteoporosis treatment rates that can easily be translated to other minimal trauma fractures.

### 5.1 Impact statement

Osteoporosis is both underdiagnosed and undertreated. Implementation of evidence‐based therapies post minimal trauma hip fracture remains a gap in post‐fracture management both in Australia and internationally. This study describes a simple and inexpensive intervention that was safe and effective in improving osteoporosis treatment rates for inpatients with hip fracture.

## AUTHOR CONTRIBUTIONS

AL: Data collection, data analysis, manuscript preparation; NS: Protocol development, data collection, manuscript review; KG: Data analysis, manuscript review; JC: Protocol development, manuscript review; LW: Protocol development, manuscript review; MJS: Protocol development, study supervision, manuscript writing and review.

## FUNDING INFORMATION

Not Applicable.

## CONFLICT OF INTEREST

The authors have no conflicts of interest to declare.

## ETHICAL APPROVAL

The project was reviewed by the (blinded) Local Health District – (blinded) Human Research Ethics Committee and was granted an exemption from further ethical review since it complied with section 5.122 and 5.1.23 of the National Statement on Ethical Conduct in Human Research (2007).
